# Tuning constitutive recombinant gene expression in *Lactobacillus plantarum*

**DOI:** 10.1186/s12934-014-0150-z

**Published:** 2014-11-20

**Authors:** Christopher Tauer, Stefan Heinl, Esther Egger, Silvia Heiss, Reingard Grabherr

**Affiliations:** Christian Doppler Laboratory for Genetically Engineered Lactic Acid Bacteria, University of Natural Resources and Life Sciences, Vienna, Department of Biotechnology, Muthgasse 11, Vienna, 1190 Austria

**Keywords:** *Lactobacillus plantarum* CD033, *Lactobacillus buchneri* CD034, Constitutive promoter, Promoter strength, Elongation factor Tu, Ribosomal binding site, BioLector™

## Abstract

**Background:**

*Lactobacillus plantarum* constitutes a well-recognized food-grade system for the expression of recombinant proteins in the field of industrial and medical biotechnology. For applications in vivo or in biotechnological processes, the level of expression of e.g. antigens or enzymes is often critical, as expression levels should be of a certain effectiveness, yet, without putting too much strain to the overall system. The key factors that control gene expression are promoter strength, gene copy number and translation efficiency. In order to estimate the impact of these adjusting screws in *L. plantarum* CD033, we have tested several constitutive promoters in combination with high and low copy number plasmid backbones and varying space between the Shine-Dalgarno sequence and the start-codon.

**Results:**

By combining strong promoters, such as transcription elongation factor promoters, isolated from *L. plantarum* CD033 and *L. buchneri* CD034, a synthetic promoter, originally derived from *L. plantarum* WCSF1 and a heterologous promoter derived from *L. buchneri* CD034 with a high and a low copy number origin of replication we demonstrated various expression levels of the model protein mCherry. All promoters were feasible for protein expression and in all cases, the high copy number origin of replication increased expression twofold. We found that the optimal spacer between the Shine-Dalgarno sequence and the start codon in *L. plantarum* consists of 8 nucleotides and elongation as well as shortening this sequence gradually down-regulates gene expression.

**Conclusions:**

We have evaluated the effects of a set of gene regulatory tools to fine tune recombinant gene expression in *L. plantarum* CD033. We have thus, provided potential expression vectors useful for constitutive protein expression in lactic acid bacteria ranging from moderate to strong production levels.

## Background

Lactic acid bacteria (LAB) are responsible for various fermentation processes leading to food and feed preservation and improvement in flavour and texture of the fermented substrate [1]. Furthermore, many LAB have been found to be beneficial intestinal microbes associated with human and animal health [2]. Thus, LAB constitute an attractive tool for many applications in food and feed production [3-5], biotechnology [6-9] and medicine [10,11]. Besides using various wild-type LAB, the possibility to expand the genetic repertoire of beneficial strains by genetic engineering becomes more and more attractive. Today, different gene expression systems are available for LAB, many of them optimized for *Lactococcus lactis* [12-15]. Inducible systems allow gene regulation by different additives such as lactose, xylose or other changing parameters like pH or temperature [16]. Another inducible expression system is based on the bacteriocin operon of *Lactobacillus sakei* which was shown to drive high-level gene expression in *L. sakei* and *Lactobacillus plantarum* [17]. Another bacteriocin induced system is the so called NICE-system (nisin-controlled gene expression system, for review see Mierau and Kleerezebem [18]), which was also adapted for use in *L. plantarum* [19]. Inducible expression systems are important when aiming at the overproduction of proteins to a maximum level, when proteins are toxic, or interfere in some other way with the host’s metabolism. LAB comprise a food grade background that by genetic engineering may be equipped with additional enzymatic activities that would be beneficial during the process of food and feed fermentation [20], for the production of food additives [6,7,21] or in the intestinal environment [22]. For these applications, inducible expression is not feasible; instead, constitutive promoters providing expression of a target gene at a suitable level are desirable. For example, the homologous lactate dehydrogenase promoter was recently used to constitutively express oxalate decarboxylase in *L. plantarum* WCFS1 [22]. It was shown in different studies, that although, bacterial promoters share similar features, promoter strength is strain and context specific and can vary significantly within LAB [23,24]. Therefore, it is necessary to identify and characterize promoters and regulatory sequences for each new host.

Besides promoter activity, also plasmid copy numbers have a major impact on recombinant protein expression. Most of the commonly used plasmid backbones are based on low copy number origins of replication (p256) [17] or high copy number origins of replication (pSH71) [25]. While in the case of ß-glucuronidase expression, a high copy number plasmid lead to increased expression as compared to using a low copy number plasmid, for a second reporter protein, aminopeptidase N, no such effect could be achieved [17]. This phenomenon was explained by gene specific properties and should be taken into consideration. Plasmid copy numbers were determined and estimated to be around three for p256 and 200 for pSH71. Another high copy number plasmid (pCD034-1) was isolated from a *Lactobacillus buchneri* strain, and its origin of replication was shown to support plasmid maintenance in *L. plantarum* [26]. The relative copy number of a pCD034-1-derivative, pCDLbu-1, was estimated to exceed 200 copies per chromosome [27]. Besides transcription and gene dosage, also translation can be a major bottle neck and design of the ribosomal binding site strongly influences protein production levels [28,29].

The goal of this study was to identify and evaluate simple tools and measures to fine-tune recombinant protein expression in *L. plantarum* with the purpose to provide suitable constitutive systems for applications in e.g. feed silage, food fermentation or in vivo drug delivery. Therefore, we compared different autologous and heterologous promoters, the impact of high and low copy number plasmid backbones and the influence of the distance between the Shine-Dalgarno sequence and the translation start signal. Our expression host was *L. plantarum* CD033. This strain has been isolated from a grass silage in Austria and may be used as an efficient starter culture for this purpose. In addition, *L. plantarum* CD033 was previously described to be feasible for highly efficient transformation with non-methylated DNA, allowing direct transfer of a ligation mix or assembled PCR fragments [30]. Therefore, intermediate hosts such as *L. lactis* or *E. coli* for high yield plasmid production are no longer required, which allows us very fast plasmid construction and manipulation, ideal for testing a large set of genetic elements.

## Results and discussion

### Comparison of promoter activities: vector design

**Figure 1:**
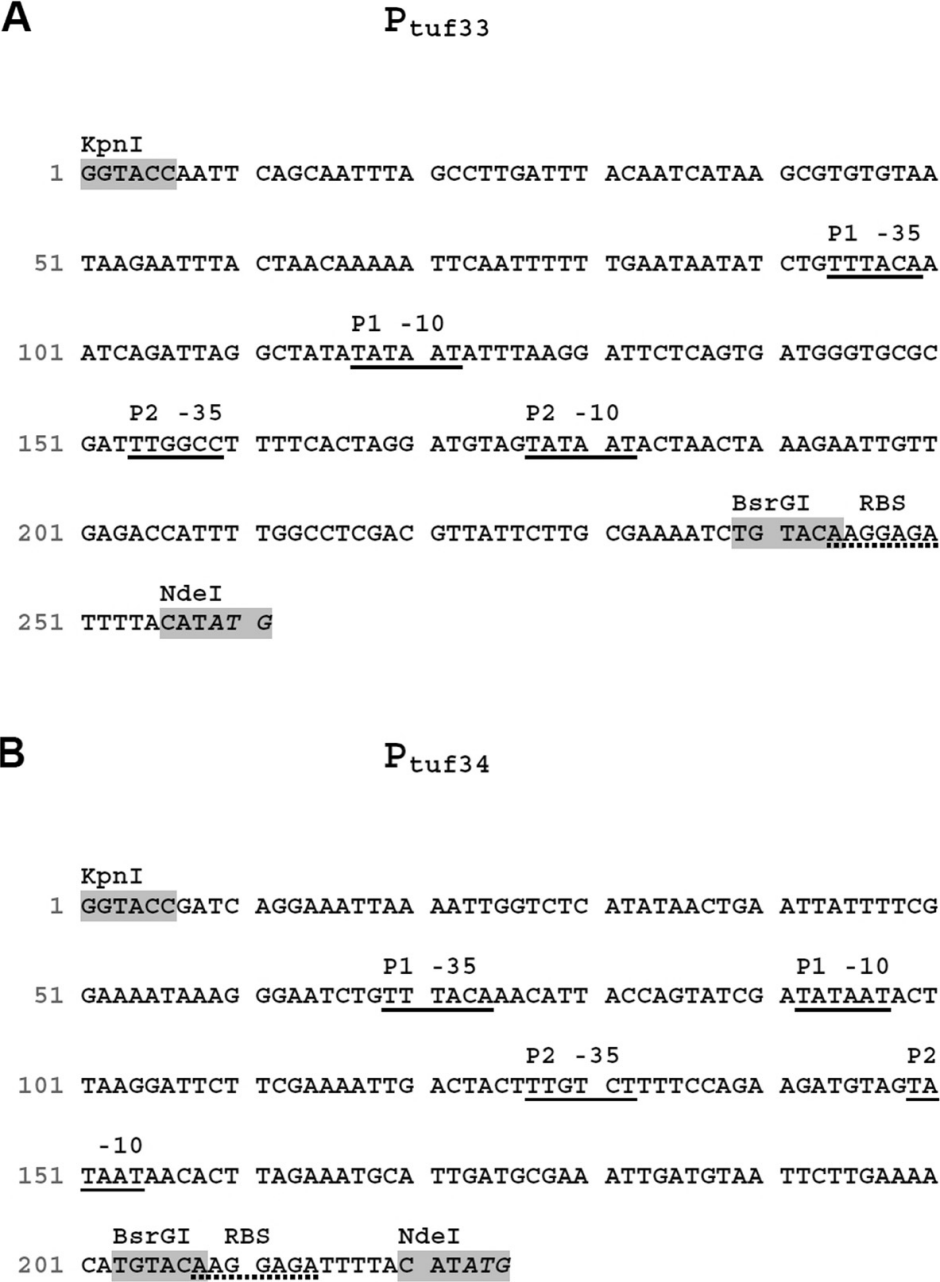
**Nucleotide sequences of promoters P**_**tuf33**_ **(A) and P**_**tuf34**_ **(B).** Both promoters are putative tandem promoters, each consisting of two consecutive promoter regions P1 and P2. The −35 and −10 regions are underlined, RBSs are underlined dotted, translation start signals are written in italics, restriction sites are highlighted in gray.

**Figure 2:**
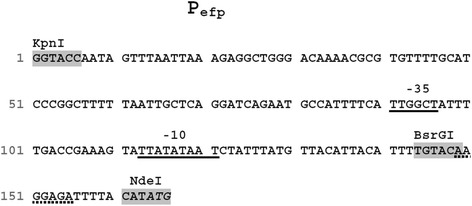
**Nucleotide sequence of promoter P**_**efp**_ **.** Ribosomal binding site, −35 and −10 regions are underlined, translation start signal is written in italics, restriction sites are highlighted in gray.

Since no amplification of shuttle vectors in *E. coli* was required, all plasmids were designed without any additional *E. coli* specific origin of replication or selection markers. In the first experiment, we included four different constitutive promoters and tested for cytoplasmic expression of the reporter gene mCherry. The strong P_11_ promoter, a synthetic sequence based on an rRNA promoter from *L. plantarum* WCSF1 [31] was previously shown to be one of the strongest promoters, active in *L. plantarum* as its transcriptional activity was comparable to the inducible pSIP-based expression system [32]. Another beneficial feature of this promoter is its cross species activity, which has been shown for *L. sakei* [31]. Further, the promoter regions upstream of the gene encoding the putative translation elongation factor TU (P_tuf_) from *L. plantarum* CD033 (P_tuf33_) and from *L. buchneri* CD034 (P_tuf34_, this study, see Figure 1) were isolated and tested. Based on the fact that transcription elongation factors are among the most abundant proteins in bacteria, our assumption was that the corresponding promoters would induce strong transcription. We compared P_tuf_-promoters from two different species in order to evaluate the versatility of this type of promoters and the feasibility for making shuttle vectors between *L. plantarum* and *L. buchneri*. In addition, using a heterologous P_tuf_-promoter would minimize possible impact by species specific regulation mechanisms. The upstream sequences of the putative elongation factor P (P_efp_, this study, Figure 2) was isolated from *L. buchneri* CD034 and based on previous observations was identified as quite active in the context of *L. buchneri* (data not shown).

### Characterization of selected promoter active fragments

**Figure 3:**
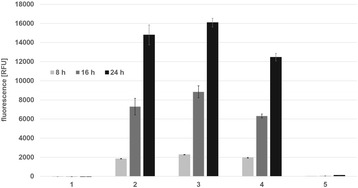
**Tecan** ^**™**^ **reader measurement of mCherry expression levels in** ***L. plantarum*** **CD033 carrying the pCD256ΔEc_mCherry vectors after 8, 16 and 23 hours.** Error bars show standard deviation. (1) *L. plantarum* CD033 negative control pCD256∆Ec_mCherry, (2) *L. plantarum* CD033 pCD256∆Ec_Ptuf33_mCherry, (3) *L. plantarum* CD033 pCD256∆Ec_Ptuf34_mCherry, (4) *L. plantarum* CD033 pCD256∆Ec_P11_mCherry, (5) *L. plantarum* CD033 pCD256∆Ec_Pefp_mCherry.

Pretesting of the promotor activities was accomplished by monitoring fluorescence signals of *L. plantarum* CD033 cells carrying the pCD256ΔEc-based constructs using a Tecan™ reader. Cells were cultivated and measurements were performed for 23 h (Figure 3). While the promoter P_efp_ was very weak, good expression could be achieved with both P_tuf_ promoters and the P_11_ promoter. The P_efp_ promoter has previously been tested in *L. buchneri* CD034 and showed medium to high expression of mCherry (data not shown), indicating that its low activity is a species specific effect, and in the context of *L. plantarum* this promoter is not feasible for further experiments.

### Impact of plasmid copy number

It has been shown for several plasmid based expression systems, that gene copy numbers have a strong influence on overall expression of a heterologous protein. While normally an increase leads to higher expression rates, sometimes too high replication rates can be detrimental to cell growth [33]. Most of the theta- or rolling circle replicating plasmids normally used in *L. plantarum* strains have copy numbers between one and five. Yet for the pSIP411-based expression system also the high copy number origin of replication derived from pSH71 is used [17]. In order to investigate the influence of plasmid copy number on the expression level, we constructed several plasmids, either containing the theta replicating, low copy number miniori p256 resulting in pCD256ΔEc plasmid constructs, or the high copy number ori pCD034-1, isolated from *L. buchneri* CD034 [26] resulting in the plasmid pCDLbu-1ΔEc. Table 1 lists all constructs used for fermentation experiments using the BioLector^™^ platform.

**Table 1:** **Description of constructs indicating the promoter and origin of replication present on each plasmid**

**Construct**	**Promoter**	**Promoter from**	**Reference**	**Origin of replication from**
pCDLbu-1∆Ec_mCherry (neg. contr.)	None	w/o promoter	This study	pCD034-1
pCD256∆Ec_mCherry (neg. contr.)	None	w/o promoter	This study	p256
pCDLbu-1∆Ec_Pefp_mCherry	P_efp_	*L. buchneri* CD034	This study	pCD034-1
pCD256∆Ec_Pefp_mCherry	P_efp_	*L. buchneri* CD034	This study	p256
pCDLbu-1∆Ec_Ptuf33_mCherry	P_tuf33_	*L. plantarum* CD033	This study	pCD034-1
pCD256∆Ec_Ptuf33_mCherry	P_tuf33_	*L. plantarum* CD033	This study	p256
pCDLbu-1∆Ec_Ptuf34_mCherry	P_tuf34_	*L. buchneri* CD034	This study	pCD034-1
pCD256∆Ec_Ptuf34_mCherry	P_tuf34_	*L. buchneri* CD034	This study	p256
pCDLbu-1∆Ec_P11_mCherry	P_11_	*L. plantarum* library	[31]	pCD034-1
pCD256∆Ec_P11_mCherry	P_11_	*L. plantarum* library	[31]	p256

**Figure 4:**
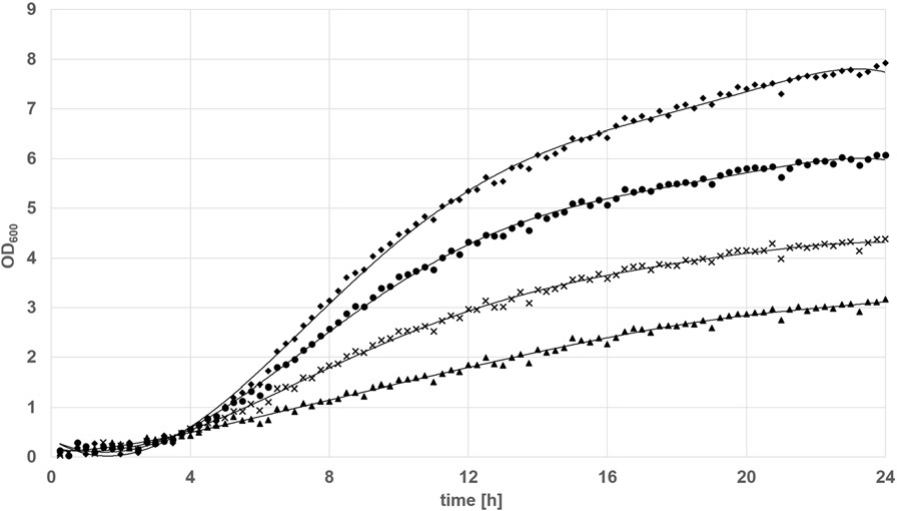
**Growth rates of** ***L. plantarum*** **CD033 carrying the pCDLbu-1ΔEc_mCherry vectors determined with the BioLector** ^**™**^ **.** (♦) *L. plantarum* CD033 negative control pCDLbu-1∆Ec_mCherry, (●) *L. plantarum* CD033 pCDLbu-1ΔEc_P11_mCherry, (**×**) *L. plantarum* CD033 pCDLbu-1ΔEc_ Ptuf34_mCherry, (▲) *L. plantarum* CD033 pCDLbu-1∆Ec_ Ptuf33_mCherry.

**Figure 5:**
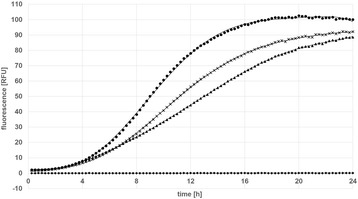
**BioLector** ^**™**^ **analysis of mCherry expression levels in** ***L. plantarum*** **CD033 carrying the pCDLbu-1ΔEc_mCherry constructs.** (●) *L. plantarum* CD033 pCDLbu-1∆Ec_P11_mCherry, (**×**) *L. plantarum* CD033 pCDLbu-1∆Ec_ Ptuf34_mCherry, (▲) *L. plantarum* CD033 pCDLbu-1∆Ec_ Ptuf33_mCherry, (♦) *L. plantarum* CD033 negative control pCDLbu-1∆Ec_mCherry.

**Figure 6:**
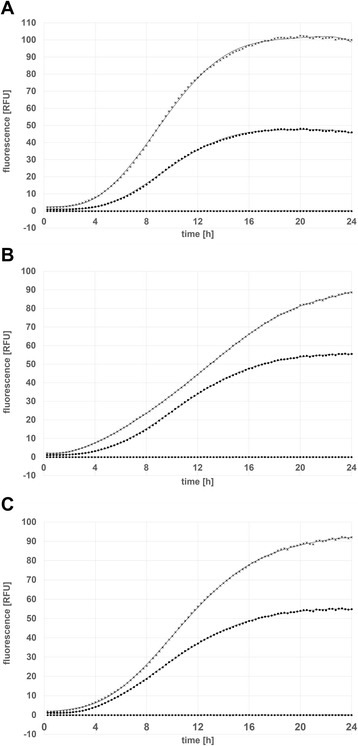
**Comparison of mCherry expression levels for the constructs pCDLbu_1ΔEc and pCD256ΔEc in** ***L. plantarum*** **CD033 measured using BioLector** ^**™**^ **platform: mCherry under control of promoter (A) P11:** (♦) *L. plantarum* CD033 negative control pCDLbu-1∆Ec_mCherry, (▲) *L. plantarum* CD033 negative control pCD256∆Ec_mCherry, (**×**) *L. plantarum* CD033 pCDLbu-1∆Ec_P11_mCherry, (●) *L. plantarum* CD033 pCD256∆Ec_P11_mCherry **(B) **P_tuf33_: (♦) *L. plantarum* CD033 negative control pCDLbu-1∆Ec_mCherry, (▲) *L. plantarum* CD033 negative control pCD256∆Ec_mCherry, (**×**) *L. plantarum* CD033 pCDLbu-1∆Ec_Ptuf33_mCherry, (●) *L. plantarum* CD033 pCD256∆Ec_Ptuf33_mCherry and under control of promoter **(C)** P_tuf34_: (♦) *L. plantarum* CD033 negative control pCDLbu-1∆Ec_mCherry, (▲) *L. plantarum* CD033 negative control pCD256∆Ec_mCherry, (**×**) *L. plantarum* CD033 pCDLbu-1∆Ec_Ptuf34_mCherry, (●) *L. plantarum* CD033 pCD256∆Ec_Ptuf34_mCherry.

**Figure 7:**
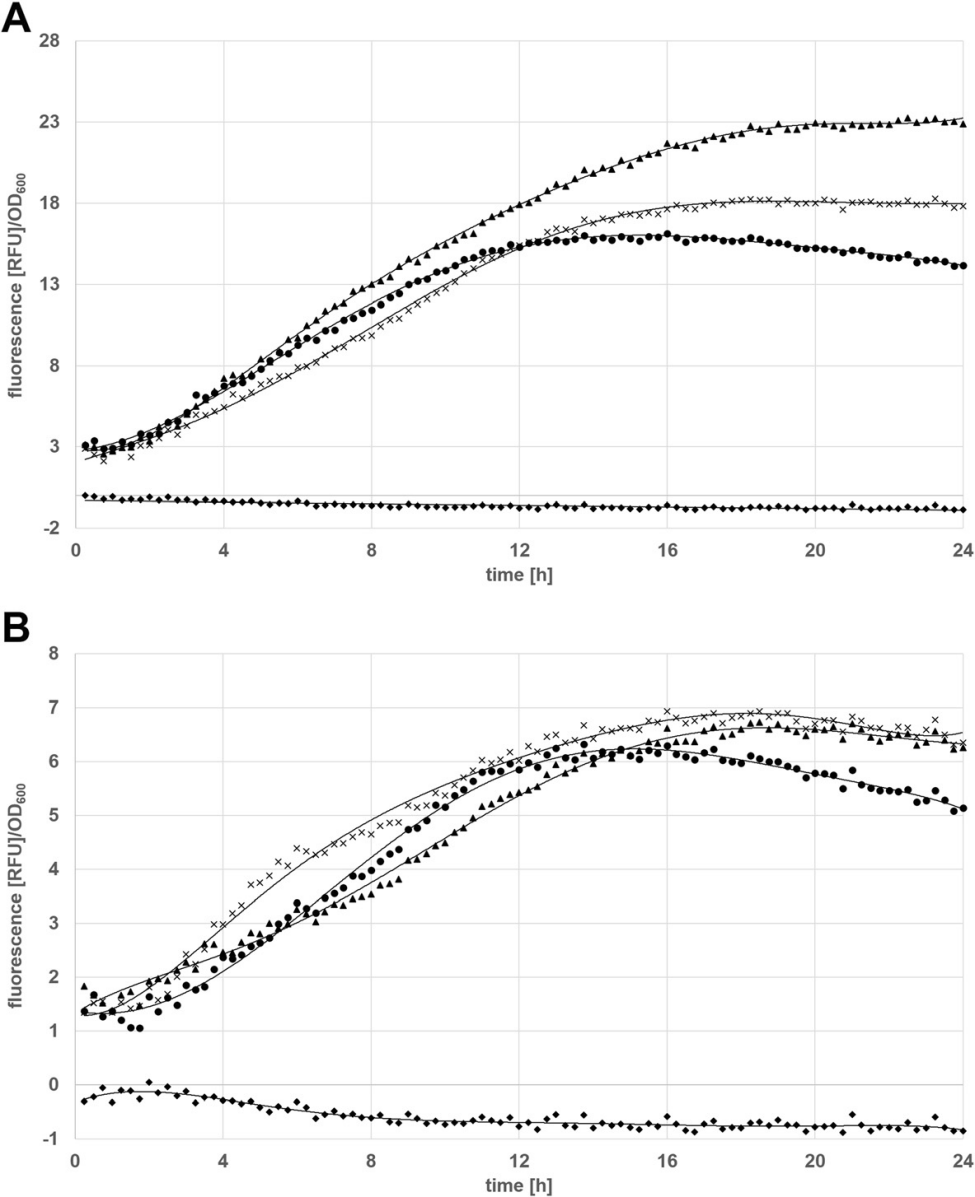
**Specific expression rates of the pCDLbu_1ΔEc and pCD256ΔEc constructs in** ***L. plantarum*** **CD033 determined with the BioLector** ^**™**^ **. (A)** pCDLbu_1ΔEc constructs: (▲) *L. plantarum* CD033 pCDLbu-1∆Ec_ Ptuf33_mCherry, (**×**) *L. plantarum* CD033 pCDLbu-1∆Ec_Ptuf34_mCherry, (●) *L. plantarum* CD033 pCDLbu-1∆Ec_P11_mCherry (♦) *L. plantarum* CD033 negative control pCDLbu-1∆Ec_mCherry **(B)** pCD256ΔEc constructs: (**×**) *L. plantarum* CD033 pCD256∆Ec_Ptuf34_mCherry, (▲) *L. plantarum* CD033 pCD256∆Ec_Ptuf33_mCherry, (●) *L. plantarum* CD033 pCD256∆Ec_P11_mCherry, (♦) *L. plantarum* CD033 negative control pCD256∆Ec_mCherry.

When looking at the growth rates of the tested clones (Figure 4), it becomes apparent that pCDLbu-1ΔEc _P_tuf33__mCherry and pCDLbu-1ΔEc_P_tuf34__mCherry, both containing the high copy number origin of replication, produce less biomass during fermentation. This might be because, due to overproduction of mCherry, the overall metabolic load hampers the growth rate. Alternatively, the high number of P_tuf_-promoter copies may capture essential sigma factors, and the cell is unable to proceed with translation of homologous genes at a normal rate. When comparing the overall transcriptional activities (Figure 5), the P_11_ promoter in combination with the high copy number plasmid backbone turned out to be the strongest, followed by the two other pCDLbu-1ΔEc-based constructs. All fermentations based on the theta replicating plasmids produced about half the fluorescence signal as compared to their rolling circle replicating counterpart (Figure 6). Growth rates were comparable for all pCD256ΔEc-based constructs (data not shown). Specific gene expression as shown in Figure 7A reflects the impact of growth inhibition in case of the high copy number constructs, while for the low copy number plasmids, specific expression rates were comparable (Figure 7B).

### Influence of the relative position of the Shine-Dalgarno sequence

The specific base pairing between the 3′-end of the rRNA and the sequence preceding an initiator AUG provides a mechanism by which the cell can distinguish between initiator AUGs and internal and/or out-of-phase AUG sequences. The degree of base pairing also plays a role in determining the rate of initiation at different AUG initiator codons in polycistronic mRNAs [34]. The ribosomal binding site (RBS) or Shine-Dalgarno-sequence (SDS) used in our expression constructs was originally derived from pSIP409. This sequence, AAGGAGA [31], however, did not correspond to the perfect matching SDS core sequence AAGGAGG, identified in *Lactobacillus plantarum*. Therefore, the RBS of the *slpB* gene from *L. buchneri* CD034, which fits better to the SDS core sequence and corresponds to the most abundantly expressed gene in *L. buchneri* CD034 was chosen for RBS-optimization (Table 2, SDOPT#9). The distance between the SDS and the start codon of our constructs comprised 9 nucleotides. For fine-tuning translational efficiency we changed the SDS to the perfect match sequence and varied the distance between the SDS and the translational start-site, analysing the range between 5 and 12 nucleotides (Table 2). All constructs were based on the low copy p256 origin of replication and mCherry expression was under control of the P_11_ promoter. The low copy origin of replication was chosen for these experiments in order to provide expression levels that can be up-regulated without causing growth hampering stress due to over-production.

**Table 2:** **List of constructs with varying spacer sequences between the SDS and the start codon**

**Construct**	**SDS-spacer-start codon**
SDOPT#5	AAGGAGG **AATAC** ATG
SDOPT#6	AAGGAGG **AAATAC** ATG
SDOPT#7	AAGGAGG **AAATTAC** ATG
SDOPT#8	AAGGAGG **AAATTTAC** ATG
SDOPT#9	AAGGAGG **AAATTATAC** ATG
SDOPT#10	AAGGAGG **AAAATTATAC** ATG
SDOPT#11	AAGGAGG **AAAAATTATAC** ATG
SDOPT#12	AAGGAGG **AAAAAATTATAC** ATG

Spacer sequences are written in bold.

**Figure 8:**
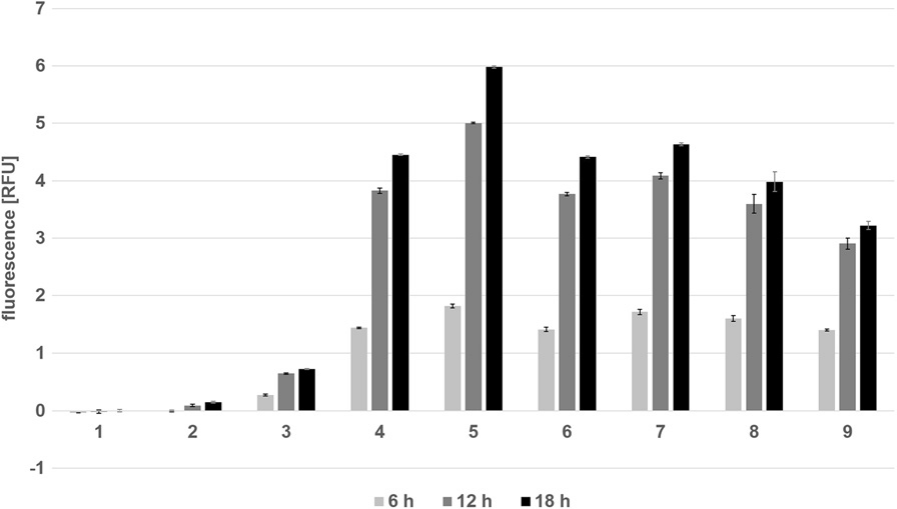
**BioLector** ^**™**^ **analysis of the SDS-varied pCD256ΔEc_mCherry constructs after 6, 12 and 18 hours.** Error bars show standard deviation. (1) *L. plantarum* CD033 negative control pCD256ΔEc_mCherry, (2) *L. plantarum* CD033 pCD256∆Ec_P11_mCherry SDOPT#5, (3) *L. plantarum* CD033 pCD256∆Ec_P11_mCherry SDOPT#6, (4) *L. plantarum* CD033 pCD256∆Ec_P11_mCherry SDOPT#7, (5) *L. plantarum* CD033 pCD256∆Ec_P11_mCherry SDOPT#8, (6) *L. plantarum* CD033 pCD256∆Exc_P11_mCherry SDOPT#9, (7) *L. plantarum* CD033 pCD256∆Ec_P11_mCherry SDOPT#10, (8) *L. plantarum* CD033 pCD256∆Ec_P11_mCherry SDOPT#11, (9) *L. plantarum* CD033 pCD256∆Ec_P11_mCherry SDOPT#12.

Growth rates were comparable for all constructs (data not shown). Figure 8 shows the correlation between differences in fluorescent signals and the varied length of the spacer sequences. The highest expression was detected after 18 h of cultivation. Spacer sequences shorter than 7 nucleotides turned out to considerably hamper translation efficiency, while 8 nucleotides seemed to be optimal. A slight decrease could be observed when the spacer was designed to be as long as 12 nucleotides. Thus, if desired, recombinant expression may be down-regulated by using spacer variations shorter than 7 or longer than 11 nucleotides. Fine-tuning of protein expression in order to utilize a host cell in an optimal way can be realized by regulation of several parameters. Here, we investigated and demonstrated the impact of transcriptional activity, gene copy number and translation efficiency for the species *Lactobacillus plantarum*. Considering that many strains of this species are used as highly beneficial starter cultures for food and feed applications, the potential applications are manifold. Over-expression of cellulases and hemicellulases could contribute to digestibility and quality of grass silage. Moreover, new substrates, such as leaves or other plant waste material could be fermented more efficiently and fed into biogas plants. Another application is the food-grade expression of enzymes for making nutritional additives, e.g. ß-galactosidase or chitinase are used to produce oligo-saccharides [6,7]. Finally, *L. plantarum* is a widely spread probiotic and as such may be used as a scaffold for vaccination or treatment of intestinal diseases in vivo. Next steps are to test different strains within the species of *L. plantarum* as well as different genes with biotechnological potential.

## Conclusions

*Lactobacillus plantarum* is widely spread in nature. It is used as a highly effective silage additive, has probiotic properties and serves as a cell factory to produce recombinant proteins. Here we have tested several constitutive promoters in combination with high and low copy number plasmid backbones in *L. plantarum* CD033. Thereby, we confirmed the previously described promoter P_11_ [31] to be feasible for strong constitutive protein expression, especially in combination with a high copy number origin of replication. We further characterized two new promoters, P_tuf33_ and P_tuf34_, which now are available as additional candidates to drive constitutive expression in *L. plantarum* as well as in *L. buchneri*. The impact of different origins of replication was investigated, demonstrating twofold higher product yields for the pCDLbu-1ΔEc-based constructs containing the high copy number origin of replication derived from the *L. buchneri* CD034 plasmid pCD034-1 [26]. Besides controlling transcriptional levels and gene copy number, we evaluated the possibility to up or down-regulate the overall target gene expression by varying the distance between the SDS and the start-codon. We could show that in *L. plantarum* CD033 there was a direct correlation between these two parameters, reaching the highest expression levels when the spacer spanned 8 nucleotides. While the performance and behavior of expression regulatory elements might differ in dependence of the target gene, predictions about their impact will facilitate vector design strategies and experimental set-ups in the future. Overall, we believe that the silage strain *L. plantarum* CD033 as well as the *L. plantarum* species in general is a highly versatile tool for improving nutrition quality, human health and biomass based energy production.

## Methods

### Cultivation and transformation of *L. plantarum* CD033

The *Lactobacillus plantarum* strain CD033 was grown in de Man-Rogosa-Sharpe (MRS) medium [35] at 30°C under oxygen limitation, supplemented with chloramphenicol (10 μg ml^−1^) if required. The transformation of plasmids into *L. plantarum* CD033 was accomplished according to the electroporation protocol described earlier [27].

### DNA techniques and cloning procedure

All Enzymes were purchased from New England Biolabs (NEB, USA). DNA fragments were amplified using the Phusion High-Fidelity DNA Polymerase according to the manufacturer’s recommendations. All resulting clones were colony screened using OneTaq DNA Polymerase as recommended by the producer. All PCRs were carried out with a C1000 Thermal Cycler (BioRad, USA). Restriction digests were performed following the manufacturer’s instructions. PCR products were purified using the NucleoSpin Gel and PCR Clean-up Kit (Macherey-Nagel, Germany). Ligations were performed using T4-ligase. All primers are listed in Table 3.

**Table 3:** **List of primers used in this study**

**Primer**	**Sequence 5′->3′**
mCherry_F (NdeI)	CAGCAGCAG**CATATG**TTATCAAAGGGTGAAGAAG
mCherry_R (BamHI)	CGTCGT**GGATCC**TTATCACTTGTATAATTCATCCATACC
P11_F (SacI,KpnI)	GACGAC**GAGCTCGGTACC**TTACAGCTCCAGATCTAGCG
P11_R (NdeI)	GACGACGAC**CATATG**TAAAATCTCCTTGTAATAGTATTT
sCAT_R (KpnI)	GCTGCT**GGTACC**GGGCAGGTTAGTGACATTAG
Tldh_F (BamHI)	CTGCTG**GGATCC**AAAACCGCTGT
mCherry_F (BsrGI)	CAGCAG**TGTACA**AGGAGATTTTACATATGTTATCAAAGGGTGAAG
Ptuf_CD033_F (KpnI)	GACGAC**GGTACC**AATTCAGCAATTTAGCCTTGATTTAC
Ptuf33_R (BsrGI)	GTCCAG**TGTACA**GATTTTCGCAAGAATAACGTCG
Ptuf_CD034_F (KpnI)	GTCGTC**GGTACC**GATCAGGAAATTAAAATTGGTCTC
Ptuf34_R (BsrGI)	GTCGTC**TGTACA**TGTTTTCAAGAATTACATCAATTTCG
efp-sense_F (MfeI, KpnI)	GCAGCA**CAATTGGGTACC**AATAGTTTAATTAAAGAGGCTGG
Pefp_R (BsrGI)	CAGCAG**TGTACA**AAATGTAATGTAACA
Cat_F (NheI)	CGACGA**GCTAGC**AATGTGGTCTTTATTCTTCAAC
M13_R (NheI)	CGACGA**GCTAGC**AGCCAGGAAACAGCTATGACC
Tldh_amp_R (PstI)	CTGCTG**CTGCAG**AAAAAGATTAAAAAAGCCGCTGC
P11_control_R (KpnI)	CTGCAC**GGTACC**CAAGGAGATTTTACATATGTTATCA
Cat_seq2_back	TACATCATTCTGTTTGTGATGG
4_6_n2_R	AACTCATAATACGCCTAAGCC
EFP_screen_back	GATTCCCGATAACAACCGT
SDOPT_5_F (XbaI)	ACGACG**TCTAGA**TAAGGAGGAATACATGTTATCAAAGGGTGAAGAAG
SDOPT_6_F (XbaI)	ACGACG**TCTAGA**TAAGGAGGAAATACATGTTATCAAAGGGTGAAGAAG
SDOPT_7_F (XbaI)	ACGACG**TCTAGA**TAAGGAGGAAATTACATGTTATCAAAGGGTGAAGAAG
SDOPT_8_F (XbaI)	ACGACG**TCTAGA**TAAGGAGGAAATTTACATGTTATCAAAGGGTGAAGAAG
SDOPT_9_F (XbaI)	ACGACG**TCTAGA**TAAGGAGGAAATTATACATGTTATCAAAGGGTGAAGAAG
SDOPT_10_F (XbaI)	ACGACG**TCTAGA**TAAGGAGGAAAATTATACATGTTATCAAAGGGTGAAGAAG
SDOPT_11_F (XbaI)	ACGACG**TCTAGA**TAAGGAGGAAAAATTATACATGTTATCAAAGGGTGAAGAAG
SDOPT_12_F (XbaI)	ACGACG**TCTAGA**TAAGGAGGAAAAAATTATACATGTTATCAAAGGGTGAAGAAG
SDOPT_R (XbaI)	ACGACG**TCTAGA**GAATACATATATGCTGGCCAGC

Restriction sites are written in bold letters.

### Construction of expression vectors for promoter activity testing

A gene, codon optimized for *L. plantarum* WCFS1 using the webtool JCat (http://www.jcat.de/), encoding the red fluorescent protein mCherry was synthesized as a gBlock (IDT, Belgium) and amplified using the primers mCherry_F (NdeI)/mCherry_R (BamHI). Promotor P_11_ was also amplified from a gBlock using the primers P11_F (SacI,KpnI)/P11_R (NdeI). The two PCR products were digested with NdeI and ligated one with each other to gain the DNA-fragment P11_mCherry. The ligation product was again amplified using the primers P11_F (SacI,KpnI)/mCherry_R (BamHI).

### Theta-replicating pCD256ΔEc-constructs

For construction of the theta-replicating expression vectors the plasmid pCD256ΔEC_hTTF1 [30] was amplified using the primers sCAT_R (KpnI)/Tldh_F (BamHI). The PCR product was double digested with restriction endonucleases KpnI and BamHI and fragment P11_mcherry was ligated into the vector backbone. The resulting construct was designated pCD256 ΔEC _P11_mCherry.

To get constructs with the other promoters, pCD256 ΔEC _P11_mcherry was amplified using the primers mCherry_F (BsrGI)/sCAT_R (KpnI). The promoters were amplified using the primers Ptuf_CD033_F (KpnI)/Ptuf33_R (BsrGI) for promoter P_tuf33_, Ptuf_CD034_F (KpnI)/Ptuf34_R (BsrGI) for promoter P_tuf34_ and efp-sense_F (MfeI,KpnI)/Pefp_R (BsrGI) for the efp-promoter P_efp_. The PCR products were KpnI/BsrGI digested and each promotor was ligated with the vector backbone described above. The resulting constructs were pCD256ΔEc_Ptuf33_mCherry, pCD256ΔEc_Ptuf34_mCherry and pCD256ΔEc_Pefp_mCherry. All constructs were introduced into *L. plantarum* CD033 by electroporation. The pCD256ΔEc constructs were colony screened using the primers Cat_seq2_back and efp_screen_back. All constructs were confirmed by sequencing using the same primers (Microsynth, Switzerland).

### Rolling circle replicating (RCR) pCDLbu-1ΔEc-constructs

For the RCR-constructs plasmid pCDLbu-1 [26] served as vector backbone. First all *E.coli*-specific sequences were removed by PCR using the primers Cat_F (NheI)/M13_R (NheI). After NheI digestion the amplicon was recircularized by selfligation and transformed directly into *L. plantarum* CD033. The resulting vector was designated pCDLbu-1ΔEC and was subsequently amplified using the primers sCAT_R (KpnI)/Tldh_F (BamHI) and digested with KpnI/PstI. The already finished pCD256ΔEc vectors served as template for insert amplification. Therefore, forward primers P11_F (SacI/KpnI), efp-sense_F (MfeI,KpnI), Ptuf_CD033_F (KpnI), Ptuf_CD034_F (KpnI) and the reverse primer Tldh_amp_R (PstI) were used to obtain the expression cassettes P11_mCherry_Tldh, Pefp_mCherry_Tldh, Ptuf33_mCherry_Tldh and Ptuf34_mCherry_Tldh. After a KpnI/PstI digest the inserts were ligated with the pCDLbu-1ΔEc backbone to gain the constructs pCDLbu-1ΔEc _P11_mCherry, pCDLbu-1ΔEc_Pefp_mCherry, pCDLbu-1ΔEc_Ptuf33_mCherry and pCDLbu-1ΔEc_Ptuf34_mCherry which were used to transform *L. plantarum* CD033 by electroporation. The pCDLbu1ΔEc constructs were colony screened using the primer pair Cat_seq2_back /4_6_n2_R. All constructs were confirmed by sequencing using the same primers (Microsynth, Switzerland).

**Figure 9:**
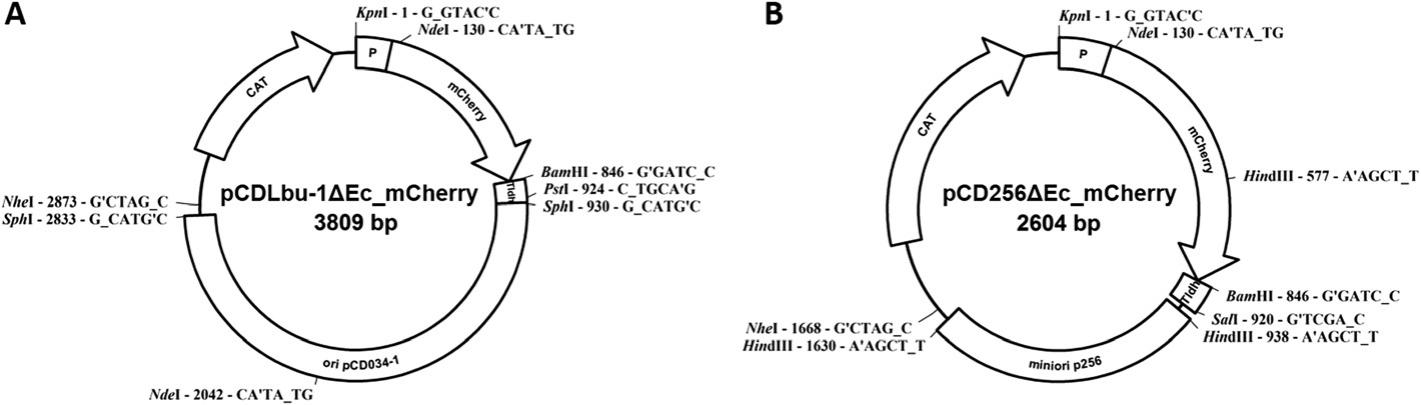
**Maps of pCDLbu1ΔEc_mCherry and pCD256ΔEc_mCherry. (A)** Map of pCDLbu-1∆Ec_mCherry consisting of the high copy replicon from plasmid pCD034-1 (Heinl et al. 2011), a chloramphenicol resistance gene for selection in LAB, the mCherry expression cassette including one of the chosen promotors, an mCherry reporter gene and the T_ldh_ terminator from *L. casei* (Spath et al. 2012b). **(B)** Map of pCD256∆Ec_mCherry containing the minimal origin of replication from plasmid p256, the chloramphenicol resistance gene, and the mCherry expression cassette.

The general vector designs are shown in Figure 9.

### Cloning of negative controls

Plasmids pCDLbu1ΔEc_mCherry and pCD256ΔEc_mCherry lacking a promoter upstream of the mCherry gene served as negative controls. Therefore, plasmids pCDLbu1ΔEc _P11_mCherry and the pCD256ΔEc _P11_mCherry were amplified using the primers P11_control_R (KpnI)/sCAT_R (KpnI). After a KpnI-digest the PCR products were self-ligated and used to transform *L. plantarum* CD033.

### Constructs for Shine-Dalgarno Optimization

Plasmid pCD256_P11_mCherry was used as PCR template for this experiment. Constructs were amplified using the forward primers SDOPT_5_F (XbaI), SDOPT_6_F (XbaI), SDOPT_7_F (XbaI), SDOPT_8_F (XbaI) SDOPT_9_F (XbaI), SDOPT_10_F (XbaI), SDOPT_11_F (XbaI) and SDOPT_12_F (XbaI) and the reverse primer SDOPT_R (XbaI). Restriction digests with XbaI were performed. The DNA-fragments were self-ligated and used to transform *L. plantarum* CD033.

Colonies resistant to chloramphenicol were screened by PCR using the primers Cat_seq2_back/EFP_screen_back and correctness of the constructs was confirmed by sequencing of the obtained PCR-products (Microsynth, Switzerland).

### Determination of mCherry expression by Tecan™ reader measurement

The Infinite M1000 Tecan™ reader connected to the Tecan i-control 1.6 software (Tecan Group Ltd., Switzerland) was used for pretesting. Overnight cultures were diluted to an OD_600_ value of 0.1. 200 μL of each sample was pipetted into a 96 well clear bottom plate (Perkin Elmer, USA). The mCherry fluorescence at 587 nm was measured at 30°C every 30 minutes over 23 h. A gain of 140 was used for fluorescence measurments. Immediately prior to fluorescence measurment, samples were shaken for 15 seconds. Samples were analyzed in quadruplicate.

### Determination of mCherry expression by BioLector**™** measurement

mCherry measurements were accomplished using the BioLector™ Basic device (m2p-labs Germany). Data were analyzed using the BioLection 2.3.13 software (m2p-labs, Germany). Overnight cultures were diluted to an OD_600_ value of 0.1 and subsequently 800 μl of each sample were pipetted into a MTP-48 FlowerPlate^™^ (m2p-labs, Germany). Fluorescence was determined using the E-OP-119 LED module for mCherry at 580 nm and a gain of 80. Measurement was executed every 15 minutes, cells were cultivated at 30°C for 24 hours under constant shaking at 1,000 rpm. Negative controls were analyzed in duplicate, samples were analyzed in triplicate. For biomass analysis a calibration curve was generated. The OD_600_ values of a *L. plantarum* CD033 o/n cultures were measured undiluted, 1:1.3, 1:2, 1:3, 1:4, 1:5, 1:7, 1:10 and 1:20 diluted in an Implen Nano Photometer (Implen, Germany) and correlated with the scattered light data at 620 nm and a gain of 20 measured using the BioLector^™^ system. The linear equation of the standard curve was y = 0.0312x – 0.6465 with a correlation coefficient R = 0.9991.

## Abbreviations

H: Hours
O/N: Over Night
OD_600_: Optical density at 600 nm
RCR: Rolling circle replicating
RFU: Relative fluorescence units
RPM: Rounds per minutes
SDS: Shine-Dalgarno sequence
W/O: Without
